# Intra-Regional Medical Tourism Demand in Malaysia: A Qualitative Study of Indonesian Medical Tourists’ Rationale and Preferences

**DOI:** 10.21315/mjms2022.29.2.13

**Published:** 2022-04-21

**Authors:** Nur Adilah MD ZAIN, John CONNELL, Mohd Salehuddin MOHD ZAHARI, Mohd Hafiz HANAFIAH

**Affiliations:** 1 Faculty of Hotel and Tourism Management, Universiti Teknologi MARA, Selangor, Malaysia; 2 School of Geosciences, University of Sydney, Australia

**Keywords:** medical tourists, preferences, National Heart Institute, Indonesia, Malaysia

## Abstract

**Background:**

This study aims to explore the Indonesian tourists’ demand for medical tourism services in Malaysia. The study also investigates the Indonesian medical tourists’ profiles and their preference for Malaysia for medical treatment services.

**Methods:**

This study conducted interviews with 49 potential patients from Indonesia who received cardiac treatment at the National Heart Institute (IJN) in Kuala Lumpur, Malaysia.

**Results:**

The findings indicate that the key motives of Indonesian tourists travelling to Malaysia for medical treatment are their disappointment with medical practices and inadequate expertise in Indonesia. Besides, they are motivated by peer recommendations, medical expertise, transparency, administration and hospitality in Malaysia. The study findings show that Indonesian medical tourists can be acknowledged as an elite group with stable and above-average income.

**Conclusion:**

Results from the study enable medical tourism marketers to better target and segments their potential medical tourists and develop a strategic medical tourism marketing roadmap. This study shows that the high demand for medical tourism is related to Malaysia’s availability of niche medical services. Besides, this paper expands the understanding of medical tourists’ decision-making and argues its implications for Malaysian health policy and healthcare delivery for the medical tourism industry sustainability.

## Introduction

The tourism industry has been identified as one of the sectors to enhance economic development. Among the popular tourism products, medical tourism was deemed a significant driver of boosting tourism, especially in Malaysia (1–3). Medical tourism in Malaysia mainly involves private medical establishments (4). Their medical treatment span includes dental care, cosmetic surgery, elective surgery and fertility treatment. Medical tourism in Malaysia has been recognised as an attractive medical tourist destination compared to other ASEAN countries (5–6). This attraction is due to the availability of well-trained medical personnel, high-quality facilities, accessible information and care, and assistance and support from government agencies and associations.

Based on the literature, the effort to promote the medical tourism industry focused on five areas. These are: i) identifying the suitable countries or markets; ii) providing tax incentives to private healthcare to develop facilities and medical expertise; iii) designing fee packages; iv) accreditation principles and v) designing advertising strategies (7–12). Moreover, the advertising strategy primarily promotes Malaysia as a ‘value for money’ medical destination and a strong regional competitor, mainly to compete with Singapore and Thailand (7–8). Other efforts include accelerating and simplifying the immigration procedure for international patients and easing the process of extending visas (4). Selected private hospitals were granted tax incentives for medical tourism promotions, including Industrial Building Allowances for building hospitals, purchasing medical equipment, staff training and service promotion (13–14).

A range of institutions such as the Ministry of Health (MOH) Malaysia, Tourism Malaysia, the Ministry of International Trade and Industry, the Immigration Department, the Association of Private Hospital of Malaysia (APHM) and the Malaysian Association of Tour and Travel Agent (MATTA) are involved in the development of a strategic plan revolving the public-private partnerships. Promoting medical tourism in Malaysia was carried out to introduce Malaysia as a hub of medical excellence (15–16). The APHM mainly assumed the professional umbrella for promoting and aiding cooperation between MOH and private hospitals (17–18). As a result, the National Heart Institute (IJN) has been recognised as the most prominent heart institute in the Asian region, specifically in cardio surgery and paediatric cardiothoracic treatments and procedures (19–20).

In addition, collaborative efforts between the Malaysian government, travel associations and the private sector attracted intra-regional medical travellers from neighbouring countries. As a result, the economic significance of medical tourism in the Malaysian marketplace is growing. Almost 60% to 70% of the inbound patients that visited Malaysia are from Indonesia, followed by other countries in the Middle East, India, China, Japan, Australia, New Zealand and the United Kingdom (21–22). The Malaysian Healthcare Travel Council (MHTC) Commercial Division Chief Officer, Nik Yazmin Nik Azman, stated that over 670,000 Indonesian nationals went to Malaysia for medical treatments and hospitality in 2018 (23). This number indicates an 11% increase compared to the previous year (24). It is estimated that around 40% to 70% of Indonesian patients come from Sumatra and Central regions (23, 25–27)

Klijs et al.’s (18) study indicate that most international patients in Malaysia are intra-regional medical travellers. Such trends prompt neighbouring countries such as Singapore and Thailand to promote medical tourism—primarily due to the perceived economic benefits of this sector (28). Notably, Indonesians prefer to travel to Malaysia for cardiac treatment (22). However, the rationale for choosing Malaysia as their medical destination was not holistically investigated. Almost three-quarters of the foreign patients in Malaysia are from Indonesia, mainly from Indonesia’s middle to high-income medical brackets (22, 29). Indonesian medical tourists exhibit diverse motivational factors in their decisions regarding treatment destinations (30–31). For example, Indonesians seeking treatment in Peninsular Malaysia are from a wealthier echelon that is more concerned with the standard of care than treatments costs (32). However, Indonesian medical tourists who seek treatments in Malaysia are more cost-conscious since some of these patients and their families opted for land transport and stayed in low-budget accommodation, privately rented homes or guesthouses or at family and friends’ homes (31). A study conducted among Indonesians from Kalimantan who travelled to Borneo Malaysia for medical treatment concluded that medical tourists sought treatment outside their countries due to poor quality of care, inaccurate medical diagnoses, frustration with hospital administrators, and limited and outdated specialised equipment (31, 33).

On the other hand, previous empirical studies of Malaysian medical tourism have shown that international medical tourists’ main pull factors were value for money, the quality of medical services, supporting services, cultural similarities and religious factors (32–34). Other studies suggest that friends, family, relatives and doctors’ referrals and attitudes of medical tourists based on positive experiences of travelling influenced medical tourists’ decisions regarding medical travel (8, 34). Additionally, a positive hospital image, accessibility, knowledge and awareness of the destination, safety and security are other motivating factors influencing medical tourists’ behavioural intention (35). In addition, the halal practices affect the satisfaction of Muslim medical tourists (36–37) and positive relationships between medical personnel and hospital services, atmosphere, facilities and quality impact the overall international patient satisfaction (38–39). However, the studies listed above focused on different profiles of international medical tourists from Southeast Asia, Australia and New Zealand and diverse medical treatments such as cosmetic and surgical procedures and medical check-ups. Besides, various factors may influence different groups of medical tourists in medical destination selection. Cross-border medical tourists may cross borders to subvert the laws of their own countries regarding specific treatments or pursue quality medical treatment (30) or gain from economic incentives of cost savings treatment (40–41).

Notably, gaps exist in understanding factors shaping patient decisions and sourcing information about the medical tourism destination. There are limited empirical studies involving primary data collection among medical tourists (42). Perhaps, this is because the demand for medical tourism is not only guided by cost considerations or clinical outcomes. Instead, medical tourism decisions are shaped by a complex matrix of motives such as perceptions of care, waiting times and cost depending on the type of treatment sought. Therefore, understanding why medical tourists choose Malaysia is crucial for developing a strategic medical tourism marketing roadmap. Hence, this study examines inbound Indonesian medical tourists in Malaysia regarding their profiles, motivation, and preferences. This is achieved through the following objectives: i) to examine the profile of tourists; ii) to identify their travel motivation factors and iii) to investigate their preferences towards Malaysian medical treatment and services. The study findings would shed light on the critical attributes of the overall medical tourist experience towards Malaysian medical tourism services.

## Literature Review

### Overview of Medical Tourism

In terms of economic conditions, medical travellers can be divided into two groups: those from high-income countries and those from low and middle-income countries (43). The group from the high-income countries is not necessarily elites in their home countries and are typically unable or unwilling to pay a substantial amount of medical charges or/or wait for the complicated medical procedure. This group belongs to the ‘global flow’ whose focus is to get a high quality of medical care, avoid long waiting periods for treatment and access affordable medical treatment. The latter groups are typically elites from low and middle-income countries that do not have access to high-quality medical treatment in their own countries. This group belongs to the ‘regional flow’ where most medical tourists are from neighbouring countries, including people in the country they are working in.

Moreover, Cohen (44) suggests four classifications of medical tourists: i) medical tourists—tourists who received treatment due to an accident or health problem encountered during vacation; ii) medical tourists proper—those who visit a particular country to receive treatment or once they arrive at the country, will decide to receive a particular treatment; iii) vacationing patients—patients for whom the main reason of travelling is to receive medical treatment, but who also take part in other tourism activities while in recovery and iv) mere patients—those who solely travel for medical treatment and do not have an intention to be involved in any tourism activities. Researchers categorised medical tourists in four categories: i) elite patients—who travel to wealthy and well-serviced cities such as London, New York and Berlin for high quality and expensive medical procedures; ii) wealthy patients—who travel for cosmetic surgery and dentistry; iii) diaspora patients—individuals that return to their homeland for a combination of economic, political, cultural and health reasons and iv) cross border patients—patients who travel seeking lower cost, faster treatment or better medical care (45–47).

Medical tourism is associated with two exits from a different perspective: duplicative and institutionalised exits (48). Duplicative exit refers to patients seeking a higher quality of medical services and specialised surgeons or avoiding waiting lists. For example, *Daily News* (49) claimed that Turkey received 700,000 international patients in 2017, mainly from Europe, Russia, Turkic republics and Gulf countries. These countries offered a short waiting time, a maximum of two weeks excluding transplantation, compared to 18 months in Western countries. Turkey is preferred for its medical specialities in eye diseases, gynaecological diseases, maternity and paediatric. In contrast, the institutionalised exit occurs when the government and other institutions set up formal programmes or policies to legalise patient mobility owing to domestic financing and/or delivery system complications. For example, the Fiji Health Ministry arranged to send their patients to India, New Zealand and Australia for radiotherapy services due to its unavailability in Fiji (50–51). Such referrals are usually excluded from discussions of medical tourism.

### Medical Tourism in Malaysia

In 1981 Malaysia adopted a neoliberal development agenda to reshape national development policy. This agenda involves encouraging corporatisation and privatisation of state assets, emphasising the importance of the private sector in contributing to the growth of the national economy through foreign direct investment in private entities. This plan has transformed Malaysia’s healthcare system into a mixed public and private system, where most of the primary and secondary care is provided by the public sector and tertiary care by the private (52). Through the privatisation of the healthcare sector, public funding was no longer available, and private financial support from insurance and personal savings became relevant under the neoliberal agenda. The Asian Financial Crisis in 1997 gave the government a further realisation of the importance of economic diversification. Thus, with the proliferation of numerous private healthcare institutions, medical tourism was encouraged to spread economic risk. Medical tourism initiative aimed to assist the private hospitals that suffered from decreasing number of domestic patients. Higher pharmaceutical and supply costs could be managed appropriately by encouraging the entry of foreign patients who were able to pay premium private hospital fees.

In an effort to accomplish the objective, a range of institutions such as the MOH, the Tourism Malaysia, the Ministry of International Trade and Industry, the Immigration Department, APHM and MATTA were involved in developing a strategic plan revolving the public-private partnerships. The promotion of Medical Tourism in Malaysia was carried out to introduce Malaysia as a hub of medical excellence (53). The APHM mainly assumed the task as the professional umbrella for promoting and aiding cooperation between MOH and private. However, some redundancy or overlap of promotional efforts occurred between the parties involved, which created confusion and wasted unnecessary resources. As a result, MOH established the MHTC as the sole agency responsible for promoting the medical tourism industry in Malaysia (54).

The commitment of the central government and related agencies to promote medical tourism in Malaysia resulted in significant growth in medical tourist arrivals from 643,000 in 2011 to 1.2 million in 2018 (55). This growth generated USD362 million of medical travel revenue in 2018 compared to USD127 million in 2011 (55). Malaysia’s cardiology, orthopaedics, oncology, neurology and dentistry services, alongside growing demand for fertility treatment, cosmetic surgery and rehabilitation services, have attracted many medical tourists (56–57). In defining the medical tourists, MHTC (21) classified them into two groups: i) health travellers (those who came for medical and healthcare purposes) and ii) foreign patients (temporary residents in Malaysia).

### Rationale for Travelling

Researchers highlighted numerous factors influencing medical travellers’ motivation in selecting their appropriate medical destination (30–31, 39, 58–61). Most available studies have summarised push and pull factors that influence patients’ decision making. Various factors such as socioeconomic status, access to health services in a tourist’s home country, cultural or psychological factors influence the decision to travel for medical assistance (56). Factors vary according to where medical services range from a simple health check-up to non-invasive treatments such as dental care to more invasive and complex procedures such as major orthopaedic operations or heart surgery (62).

The long waiting lists for medical treatment are a common reason why medical tourists seek treatment outside their home countries (63). Public healthcare often involves long waiting times, feeding a demand among patients for fast and effective treatment. In addition, the government’s treatments not recognised as a priority and not covered by insurance lead to long waiting times and limited consultation times that also raise concerns on the validity of the diagnosis. A long waiting list, especially in receiving an organ transplant, complex medical restrictions and high costs encourage more patients to seek treatment alternatives abroad (64). Among medical tourism countries, the Philippines is the only destination that promotes packages offering all-inclusive organ transplants (65), although illegal in other countries. Long waiting times for general medical services in other countries and a lack of insurance support influence medical tourism’s success.

On the other hand, medical cost influences their decision to opt for medical tourism destinations. The term ‘first-world health care at third-world prices’ has long been emphasised regarding medical treatment in low and middle-income countries (66). Countries with facilities that advertise inexpensive health procedures are attractive to lower-income medical travellers. This situation is primarily the case with dental treatment since public health insurance does not cover it. Thus, travelling to countries that provide comparatively low prices is one of the few available options for accessing affordable medical care (67). Countries like Malaysia, Thailand and India offer affordable and reasonable accommodation, food and beverage and local transport cost. This cost-saving factor is attractive to medical travellers who are either underinsured or must pay for procedures that require them to pay out of pocket (4). Due to the exorbitant cost of health services in high-income countries, lower medical costs offered by low and middle-income countries are an influential factor (68).

In another perspective, inadequate quality of medical services and unavailability of treatment are the factors that encourage medical tourists to seek treatment abroad (69–70). Based on the Nigerian Sovereign Investment Authority (NSIA), Nigerians spend more than USD1 billion on medical tourism annually for various medical treatments. These treatments include oncology, orthopaedics, nephrology and cardiology treatments. The low level of confidence in health professionals (HPs)’ competence and inadequate medical infrastructure are the main factors that motivate Nigerians to seek medical treatment abroad (71). The Cayman Islands are another example of a state’s inability to provide quality tertiary care, leading to the islands’ movement, primarily to the United States. This situation resulted in USD30 million spent per year on healthcare overseas (72). The growing epidemics in low-income countries place a tremendous burden on countries with inadequate health systems. In Asia, Laotians seek medical treatment across the Thai border due to Laos’s inadequate medical facilities (72). Moreover, limited local expertise and outdated facilities encourage Indonesians to cross the border seeking adequate medical care after local treatment has proved inadequate (30–31).

Besides, cultural similarities may influence the choice of destination. Geography, culture, language and religion play roles whereby medical tourism destinations cater to specific populations of medical tourists who prefer to obtain medical procedures in a familiar culture and background (73). Language and cultural factors also attract diaspora to return to their home countries to receive medical treatment (74–76). For example, Korean migrants who are permanent residents in New Zealand or New Zealand citizens often return to Korea for medical services. Comfort in language, cultural factors and familiarity with the healthcare system encourages them to return to their homeland to access healthcare services. This is also inspired by the availability of family and friends in Korea to assist with the medical trip (76).

For example, in some countries within the European Union, some patients opt to be sent abroad to receive medical care under bilateral and multilateral agreements. These patients are outsourced patients and often travel relatively short distances to the specific hospital that fall under the arrangement due to inadequate specialists and facilities in the home country (77). Gulf Cooperative Council member states also send citizens to hospitals in Thailand for medical treatments in neurology, orthopaedics and oncology (78). Outsourcing patients to other countries for medical services is a cost-effective strategy among wealthy countries by relieving the treatment cost to insurers. Insurance agencies can provide cheaper insurance packages by transferring the patients to international destinations with cheaper labour costs. However, in Europe, most cross-border medical travellers decide to travel individually rather than under government arrangements (79).

Furthermore, medical travellers also often seek illegal medical services in the country of origin, for example, transplant procedures or assisted suicide (80–81). The desire to receive unavailable medical treatments that are new and/or difficult or illegal often leave seeking treatment overseas as the only available option. Some medical establishments promote and offer high risk or experimental procedures not available in other countries, such as stem-cell treatment, to widen the range of medical options offered and attract more medical tourists (38). Other medical tourists may travel to avoid laws and regulations surrounding certain procedures at home, such as abortion, infertility treatments and surrogacy arrangements (82–83). Many lesbian couples and single women in France, for example, cross the country border to Belgium specifically to receive donor sperm as their home country denies access to such treatments for same-sex couples (84). This movement is recognised as ‘circumvention tourism’ as patients travel abroad to circumvent their home countries’ laws (85).

Last but not least, the need for anonymity motivates them to travel outbound for medical care. Confidential care is a priority for many patients concerned about disclosing their health information when receiving treatment in their home countries. Medical services or treatments like drug rehabilitation, gender transformation surgery or plastic surgery often involve individuals who prefer privacy and confidentiality (86). As distance provides anonymity, receiving medical care away from home offers anonymity and confidentiality that might otherwise be unavailable (40). For example, many Japanese medical tourists travel to South Korea to receive cosmetic treatment in a country known for strict confidentiality of patient records, alongside South Korea’s reputation for surgical skill and lower medical costs (47).

### Decision-Making Process

**Figure 1:**
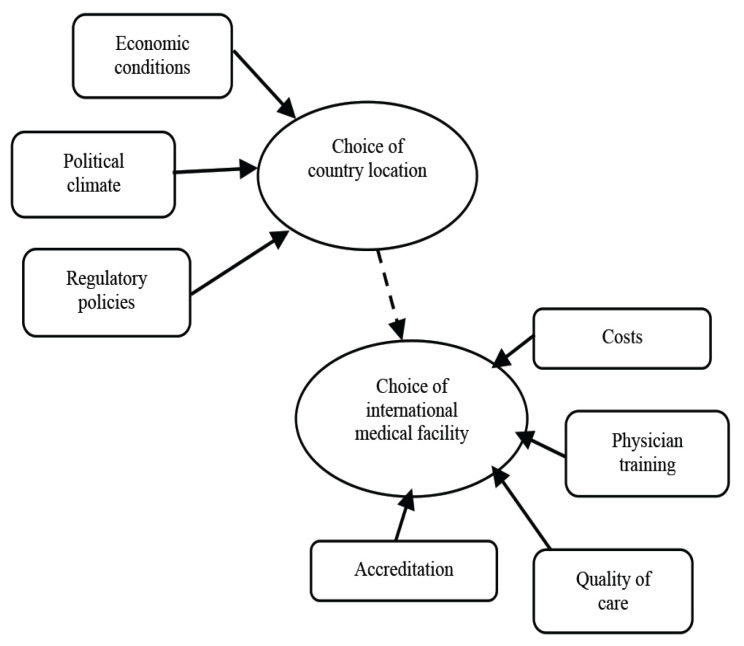
Two-stage model of factors that influence medical tourism decision

The push and pull concept emerges when considering what motivates people to travel for medical tourism (46, 87–89). It identified a two-stage model of medical tourism decision. Firstly, the choice of country location and secondly, the choice of medical facility, both driven by push and pull factors (89) (Figure 1). Smith and Forgione (89) mentioned that no dominant factor influenced patients’ decision making and various factors contributed to influencing the decision to travel to seek medical care and treatment. They added that once a patient has considered the medical destination, they must consider the healthcare services offered, including specialised procedures, high technology equipment, and good physicians and specialists. However, this model only focused on explaining the range of factors that encouraged medical tourism but neglected to explore other dominant factors for each individual which ultimately influence their final decision.

**Figure 2:**
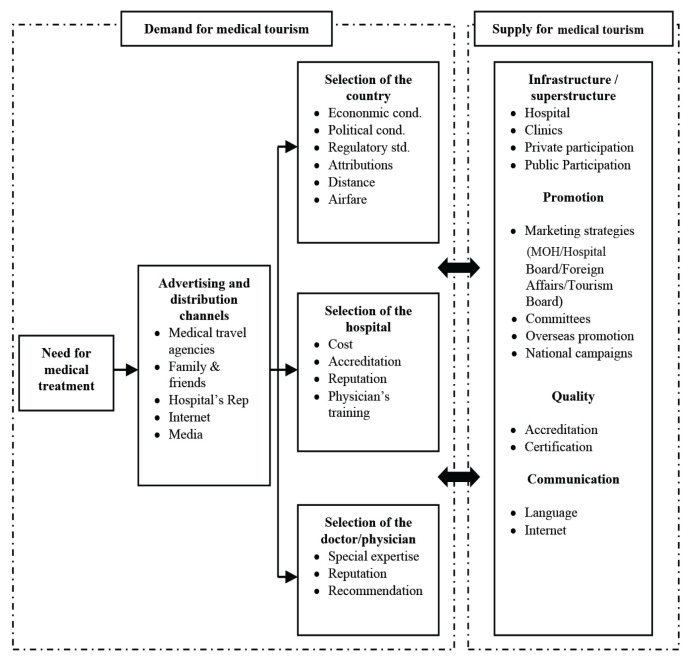
Conceptual model of medical tourism

Heung et al. (90) developed a more detailed conceptual model of medical tourism (Figure 2) that consists of supply and demand considerations. Demand refers to factors that encourage patients to seek medical treatment overseas. Supply refers to attributes offered by the destination in fulfilling the demands of medical tourists. However, this conceptualisation does not focus on what medical tourists face, encouraging them to seek treatment abroad. The model does not explain what factors stimulate or force medical tourists to leave their country to seek medical treatment. In short, while push and pull factors are valuable in shaping medical tourism decisions, decisions go beyond basic individualistic aspects. Consequently, questions remain as to whether existing medical tourism decision-making concepts apply to Indonesian medical tourists in Malaysia. Various factors such as individual needs, the evaluation of destination and medical facilities, the location of kin and friends, the influence of medical facilitators and the internet are all part of medical tourism decisions, emphasising the complexity of medical tourism decision making (91).

## Methods

### Research Design and Population

The qualitative case study approach through the interview was applied for information gathering. The population were among the Indonesian patients who received cardiac treatment at the IJN, Malaysia. The Indonesian patients and companions were chosen based on their availability and willingness to participate in the study. The interview questions were mainly developed from the existing literature and the questions employed must fit the research objective. Preliminary interviews were undertaken as the basis for refinement. The open-ended questions were used to avoid any potentially biased responses and with the idea to discover rather than prescribe.

### Instruments

Questions ranging from demographic profiles, the experience of receiving treatment in their own country, and the reasoning of choosing Malaysia were probed. Two versions of semi-structured questions (English and Bahasa Malaysia) were used. The original version was translated into the Malay language by a language expert. Questions were carefully worded to allow informants to understand and stimulate the conversation. Any grammatical errors or language comprehension errors in both language versions were corrected. This study received ethical approvals from the University of Sydney before fieldwork. Besides, permission and cooperation from the IJN were obtained. Since researching patients involves restricted and sensitive circumstances dealing with patient confidentiality and recuperation, support was required. It is also had the potential to create discomfort among patients. Once an official approval from IJN and complied with the enforcement of the Personal Data Protection Act (PDPA), the dates and times of the interviews were then arranged based on the patients’ convenience and wishes, which required unlimited flexibility on the part of the researcher.

Before data collection, a pilot test was conducted among three selected Indonesian colleagues who have travelled to Malaysia for medical tourism to ensure that the interview questions were understood. According to the pilot test results, several redundant questions caused the informants to give the same answer. Another point raised is that the number of questions is excessive and thus needs to be adjusted and the focus narrowed appropriately. The feedback was refined based on the information gathered from the pilot test and incorporated into the final draft of the semi-structured interview questions. It is worth mentioning that, before the interview session, the informants are explained that their participation is voluntary, all the information provided is strictly confidential and that their names will not be revealed. Most of the interview sessions are in Malay language except for a few, and all of them were tape-recorded. On average, each interview lasted between 40 min and 1 h. Despite some issues on the willingness to participate, the interview sessions were successfully undertaken. Interviews were conducted until a saturation point was reached where no new information was obtained. The numbers of respondents in this study were not predetermined but depended on data saturation. The data saturation was verified when repeated feedback was evident in the last few interviews.

### Data Collection

Approaching medical tourists and their companions at the hospital was complicated by patient health conditions and personal and family privacy. Snowball sampling provided a means of building trust with participants. The IJN expressed concern that conducting interviews might create an uncomfortable environment and interfere with patients’ and families’ privacy. Hence, in conducting the interview sessions, the hospital administration determined which patients could be approached only after attaining patient consent. The hospital administration is the front-line contact that administers and communicates with the patients and their companions during the registration process. They are competent in disseminating information and organising the flow of patients for health screening examination before the doctor’s consultation. When the hospital administrator permitted interview sessions, the patients were free from health examinations but simply waiting for their turn for the doctor’s consultation. This waiting time was the best time for the interview sessions as they were not involved in other health examinations or tests. A total of 49 interviews were undertaken. The duration of the interviews ranged from 20 min to 40 min. Interview sessions were conducted between September 2019 and December 2019. The hospital administrator determined which patients to interview after getting their daily consent.

### Data Analysis

All of the responses from interviews were transcribed verbatim. Responses were transcribed with the exact words used, and all the critical incidents were described. For Bahasa Malaysia responses, data was first written in Bahasa Malaysia then translated into English. Written English responses were then compared with the Bahasa Malaysia responses to ensure data had been accurately translated. The first analysis stage involves transcribing each interview and making notes in a reflexive diary. Next, the researchers use descriptive data for topic analysis and then identify, analyse, and report pattern codes. The coding process was manually done and followed by qualitative data analysis using ATLAS.ti version 8. This study uses narrative analysis when interpreting the interviewed information. It is worth mentioning that each name of the informants is not disclosed to preserve confidentiality. After data transcription, data analysis involved interpreting collected data to reflect the research inquiry’s interests, ideas and theories. Thematic analysis was chosen to identify, analyse, and report patterns or themes captured from the interview data about the research questions. Researcher judgment was important to determine what theme emerged from the data set, and flexibility was required in managing the theme. To assure content validity, inter-rater reliability tests were assessed and found acceptable with a Kappa score of 0.78.

In thematic analysis, there are two primary methods for identifying themes or patterns within the data set known as: i) inductive or bottom-up and ii) theoretical, deductive or top-down. The inductive approach means the recognised themes are strongly linked to the data collected. Therefore, the themes are not driven by the researcher’s theoretical interest or literature review. Therefore, the coding process does not attempt to associate themes with pre-existing codes or analytic preconceptions. The data is re-read to identify themes related to the focus of the study, and coding is performed based on themes discerned in the data. Interview questions and themes were based on the available literature to enable a better degree of comparison and complementarity with earlier studies.

## Results and Discussion

### Informant Profiles

All informants were married, and the majority were males of the late middle age group. This finding is unsurprising as cardiovascular risk factors were more prominent in elderly patients and higher in men than women. Medical companions were often the medical tourists’ spouses and children; hence, most companions were married. Most medical tourists and companions were from middle to senior age groups. More than three-quarters of the medical tourists and their companions were from the capital region, Sumatra and Java regions. Most lived in the largest cities. The majority of them and their companions were well educated and had a minimum senior high school education. Few had only junior high school qualifications. Most of them were Muslim, with a small number of other religions. Finally, medical tourists with their companions stayed in Malaysia for a minimum of one day to more than seven days.

### The Rationale for Travelling to Malaysia for Medical Treatment

**Figure 3:**
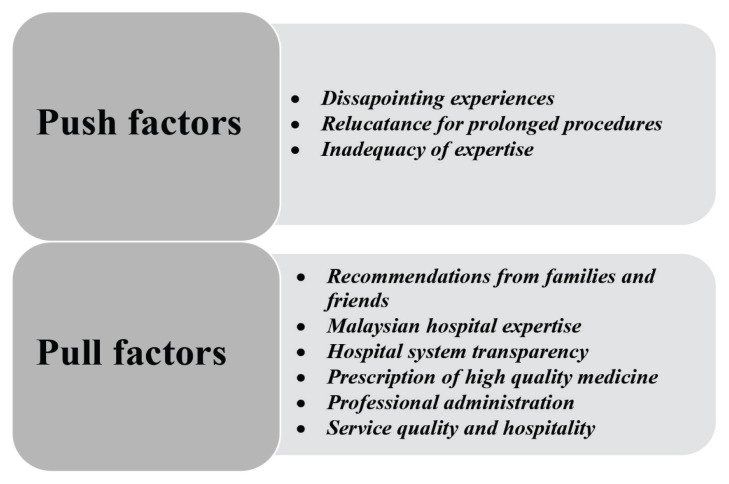
Research findings

This section focuses on medical tourists’ rationale for travelling to Malaysia to receive cardiac treatment. Two themes have been identified through the description: the push and pull factors. The push factors refer to the experiences faced by medical tourists and their companions that influenced them to leave their home country. Meanwhile, the pull factors refer to matters that led medical tourists to seek cardiac medical treatment outbound, specifically in Malaysia. Figure 3 depicts the study findings graphically.

#### Push Factors 1: Disappointing Experiences

Most responses indicate that they received treatment in their country several times before finding another medical option. Disappointing experiences such as complicated medical procedures and administrative inconvenience, unnecessary medication, long waiting time, incorrect diagnosis and slow health improvements make them pursue medical treatment in Malaysia. All of these factors contributed to frustrations and disappointments. A few informants (Informant 1, Informant 3 and Informants 5) stated that even though they had received medical treatment for a long time at the same hospital, complex medical processes and unnecessary requirements resulted in frustration and disappointment with the hospitals. Most of them felt unimportant and complained of inadequate attention in the hospital system, thus encouraging them to opt for medical treatment elsewhere. Informant 6 and Informant 7 shared similar views. They were required to undergo heart valve surgery, waiting for their critical condition over the last two years. Due to long waiting lists, the Indonesian-based hospital postponed their surgery a few times and prescribed various generic medications. They found the prescriptions unhelpful as they felt there had been no progress on their condition. Informant 9 noted that a long queue for specialist consultation was detrimental to his health. This situation was compounded by disorganised medical records, which eventually resulted in his decision to find better medical alternatives.

#### Push Factors 2: Reluctance for Prolonged Procedures

Conflicting medical advice and treatment received by medical tourists before visiting IJN led to confusion and negative perceptions of the country’s hospital credibility. It was claimed that some hospitals were making money through prescribing several varieties of medicine and recommending possibly unnecessary and potentially high-risk surgeries. Inflated medical costs contributed to negative perceptions and distrust and encouraged the informants to search for other alternatives. Ten informants receiving treatment in IJN reported that they were reluctant to have procedures in their country hospitals, thus seeking further medical advice and low-risk alternatives. They went to IJN after receiving medical advice from two different hospitals in Indonesia. Meanwhile, Informant 11 and Informant 12 stated that the decision to visit IJN resulted from his doctor in one province’s city deciding to conduct heart surgery without being fully assured of his actual condition and illness. Other reasons, including seeking a better alternative to surgery based on a recommendation from relatives, was highlighted by Informant 14.

#### Push Factors 3: Inadequacy of Expertise

The inadequate expertise and lack of cardiac medical services in Indonesia encouraged most medical tourists to consider receiving medical services in other places. Informant 15 and Informant 20 claimed that having received long-time medical treatment and ineffective medication prescribed in one of the hospitals in their province make them opt for Malaysia medical providers. In addition, they also claimed that the unavailability of expertise in the home country led them to choose IJN for bypass surgery services. Informant 22 accentuated that effort to find other hospitals in his own country capable of conducting the procedure was in vain due to the lack of expertise, hence refusing to treat him. Without any other option, it had directly encouraged him to seek medical treatment in Malaysia.

#### Pull Factors 1: Recommendations from Families and Friends

Based on the interviews, friends’ past experiences, family recommendations, and local doctors’ recommendations influenced the informants to choose Malaysia for further medical treatment. Besides, their past experiences and information gathered from trusted and familiar people reduce their uncertainty and provide assurance about the outbound medical services. Some medical tourists had visited other hospitals in Malaysia but chose IJN due to friends’ recommendation to consider it for a long time. In other words, others’ success stories related to the outcome of medical treatments cause the causation. Informants 23 and 24 stated that positive feedback from a friend attracted them to visit IJN for a full heart screening and examination.

In contrast, Informant 30 revealed that family recommendations encouraged him as a first-time medical tourist to choose IJN. These recommendations were from kin who lived in Malaysia or other family members who had visited IJN before. As medical tourists, Informant 31 and Informant 40 noted that none of their family members or friends had received medical treatment outside Indonesia. Thus, without such guidance on Malaysia’s medical system, their decision to visit IJN to receive medical treatment for the first time was mainly influenced by their Indonesian doctors. The latter advised them to find another medical alternative. A high standard and quality of care are reasonable concerns before travelling to another country for medical services. These relevant areas of specialisation could serve as a magnet for drawing informants in seeking specific medical treatments, uttered by Informant 43.

#### Pull Factors 2: Malaysian Hospital Expertise

The study found that the hospital expertise and specialisation influence medical tourists’ trust and confidence. This finding is evident in their continued visits for routine follow-up sessions, sometimes over several years. Although few visited other hospitals in Malaysia (occasionally Pulau Pinang and Melaka), medical tourists eventually altered their medical treatment destination to IJN due to its expertise, especially in heart-related disorder treatments. Information platforms in disseminating information about IJN are from Indonesian hospitals and Malaysian hospitals and online resources such as hospital websites, online news, and articles. For Informant 29 in particular, the superior quality of medical treatment, technology or care offered in another country is a key rationale for him travelling outside their home country for medical treatment. For informant 27, Informant 44 and Informant 35, IJN is seen as a reputable provider of specific cardiovascular treatment and services through their pool of experts and experienced medical personnel has positioned the hospital competitively nationally and internationally. Moreover, medical expertise is one of the major factors influencing them to revisit IJN.

#### Pull Factors 3: Hospital System Transparency

Transparency is the condition of being transparent. The hospital system transparency is related to openness, communication and accountability of hospital practice. It is associated with a clear explanation of the medical tourists’ health conditions, prescribing medicines essential and giving a precise cost of the treatments. The integrity, openness, and consistency in providing medical services influenced the informants’ trust and, ultimately, the particular medical services. The absence of transparency discourages medical tourists from using their local hospitals.

Informants 36 and 17 had visited IJN for medical health observations and check-ups for almost four years. They stated that the medical practices at IJN, notably in providing time to explain their health status, was in-depth, transparent and more valuable than anything available in their country. As a medical tourist, Informant 18 commented on doctors’ transparency practices in IJN in explaining his condition and the needed treatment. Before bypass surgery, the doctor would explain the procedure processes, success rate and risks which eased his and his family’s concerns.

#### Pull Factors 4: Prescription of High-Quality Medicine

The effectiveness and availability of medications at IJN somewhat influenced medical tourists to revisit. The availability of specific medical services in the visited country suitable for informants’ conditions influenced their repeat visits. For IJN, as stated by some medical tourists (Informant 34 and Informant 13), the hospital’s quality of prescription medicine and their perceptions of the outcome directly influenced them to revisit it. For Informant 25, she visited IJN for six years as he could not find expertise in their local hospital. Informant 23 stated that the medication at IJN was more effective than medication in his home country. Similar points were made by Informant 21 and Informant 38, who were receiving medical follow-up sessions at IJN with ongoing visits to the hospital aimed at obtaining prescriptions.

#### Pull Factors 5: Professional Administration

Undeniably, systematic and well-arranged hospital information and activity flows are essential for informants. Regarding the medical examinations and consultations that could be completed in a day, the organised administration system was a key element that smoothed and simplified the treatment processes and encouraged medical tourists to choose IJN. Informant 8 stated that IJN adopts a systematic, integrated hospital administration network. From the registration to the medical test, doctor consultations and medicine claims were orderly and attended adequately by medical personnel. The same issue was mentioned by Informant 4 as at IJN. All the medical examinations and consultations could be completed in one day. He added that at IJN, the medical personnel would provide him with an update of the doctor’s status if there was any delay while waiting for consultation. Informant 38 revealed that he could establish communication with the hospital administration personnel via phone and email his medical report to make a medical appointment. Later he would be given a suitable doctor and time appointment.

#### Pull Factors 6: Service Quality and Hospitality

The hospitality provided by IJN staff through attentiveness, consideration and friendliness made medical tourists and their companions feel appreciated. For informants, such hospitality is important. These supportive medical services made a significant difference in their perceptions of the hospital services. Thus, the choice of location for care. Hospitality is related to meeting guests’ needs through a positive host and guest relationship in commercial operations. Consequently, the hospital plays a role as a host in providing needs through the medical services experienced by their informants.

Informant 26 and Informant 39 espoused the hospitality provided by the medical personnel as part of the reasons that influenced his decision to receive medical treatment for several years at IJN; the openness and friendliness of the doctor who treated him made his visit to the hospital more welcoming. Informant 27 recounted a similarly positive experience, noting that IJN medical services were beyond his expectations. He mentioned how medical personnel attentiveness and care in delivering medical treatment were positive in contrast to what happened in hospitals in his province where the informants seeking medical treatment under insurance coverage experience poor services.

## Implications

The findings of this research can contribute to medical tourism policy formation. One of the main initiatives taken by the government in promoting medical tourism in Malaysia is focusing on high-quality medical treatment. Similarly, other studies propose the ease of travelling across the land border, supported by easy communication through understandable explanations of health conditions and medical expertise. Most Indonesian patients claimed that Malaysia offered professional medical services using this study’s latest equipment and technology. Therefore, promoting high-quality medical care services by emphasising cultural aspects, such as the similar language and Asian ethnic cuisine, made Indonesian travellers feel welcome. While important for Muslim Indonesians, these elements could also attract medical tourists from other Muslim markets such as the Gulf countries and Bangladesh.

These revelations carry varying consequences and implications to the relevant authorities, particularly the MOH, MHTC, the Tourism Malaysia, public and private hospitals. Besides providing medical services, medical tourism can boost local revenue and stimulate economic growth. In addition, medical tourism presents an opportunity to attract international patients and offset the high costs of healthcare provision and associated pharmaceutical expenses. Essentially, offering medical services to foreign patients is economically beneficial and commercially profitable for many low and middle-income countries.

This research study’s contribution lies through a deeper and more comprehensive intellectual understanding of the medical tourism demand in the Malaysian context. With such information, it creates awareness amongst relevant parties such as hospitals, clinics, travel agencies and government authorities, especially the MOH, MHTC and the Ministry of Tourism, Culture and Arts to improve, maintain and upgrade the current and future structure of medical tourism in Malaysia. Besides, the local community, hospitals and clinics involved with international medical patients may benefit from the findings by improving, maintaining and increasing the products and services offered. This will further help travel agencies have general safety guidelines to promote and induce Malaysia’s positive and relevant image for general and medical tourism, especially once the border reopens.

More importantly, given the post-COVID-19 era, to build confidence and greater acceptance of international patients on the competence of healthcare providers, a clear direction on the medical tourism instrumentation must be underlined and highlighted. For MOH and the Ministry of Tourism, Culture and Arts responsible for medical tourism, the findings from this study will aid them in scrutinising the drawbacks and strengths of their policies and marketing strategies for the overseas market.

## Study Limitation

This study focuses on a group of high-income medical tourists seeking cardiac care, which implies innate and obvious limitations. A more in-depth analysis could be conducted on another group of Indonesians (including those who have not chosen to travel to Malaysia) or other international patients of different levels of socioeconomic profile to understand the diversity of medical treatment requirements. This may capture a more holistic understanding of the medical tourism pattern and structure, thus further insights into its impact on the local and national economy or the public health care sector. Besides, this study revealed that Indonesian medical tourists and their companions participated in tourism activities during their stay. However, whether these activities are also similarly undertaken by other medical tourists elsewhere in Malaysia. Thus, comparative analysis in different states would provide a more comprehensive understanding of other tourism activities and expenditure patterns. A limitation of this research is that it mainly focuses on Kuala Lumpur and therefore provides a partial understanding of the tourism activities of Indonesian medical visitors. A comparative analysis between other states could offer data on the profile of other medical tourists of different categories and national origins. This would give a more comprehensive understanding of medical tourism.

Meanwhile, with the current COVID-19 pandemic situation, it is difficult to guarantee that patients receive the required level of care. The current condition of the COVID-19 pandemic threatens the stability of the country’s medical tourism sector as it affects the ability of people to travel. With the increasing number of people infected, almost all international borders are expected to be closed potentially beyond 2022. Hence, travelling regularly to medical tourism countries for ongoing medical consultation and treatment will be more difficult, as border closures may limit their movement. The movement control policy resulted in further uncertainty and challenges in the future of the medical tourism industry related to the complicated hospital procedures for accepting international patients and complex requirements for crossing the international border. As Malaysia faces many COVID-19 cases locally, things will no longer be the same. Hence, the study findings are limited to medical tourists’ future perception of Malaysian health management and would change how medical tourists consider Malaysia.

## Conclusion

The findings show that the Indonesian medical tourists were relatively educated, with 90% receiving tertiary level education. They were in middle-upper and high-income categories based on their occupation background. Most of them had a reliable source of income, were involved in trading, ran their businesses, and were professionals in various fields. They chose to have treatments in Malaysia even though it was a relatively expensive medical and surgical treatment choice. Therefore, this group of medical tourists can be acknowledged as elites from a developing country with stable and above-average income. The basic rationale for travel was receiving adequate cardiac treatment. However, that was influenced by multiple secondary factors. Medical tourists sought treatment outside their own countries due to less quality of care, inaccurate medical diagnoses, hospital administrators’ dissatisfaction, limited specialised medical expertise and less advanced equipment. The Indonesian medical tourists relied on recommendations and information from family and friends to choose IJN among first-time medical tourists. The Indonesian doctors also constitute a new source of information in this study. The role of Indonesian doctors in recommending patients consulting them to get further treatment in Malaysia, particularly at IJN, followed by personal contact from attending conferences and medical discussions, alongside positive feedback from former IJN patients under their consultation.

In addition, as first-time medical tourists with no experience or little information about IJN, other information platforms such as YouTube, online news and articles, the hospital website and hospital representatives in Indonesia were used to strengthen their confidence. These information platforms were considered important tools to create awareness and interest among first-time medical tourists unfamiliar with Malaysia’s medical services. Online or virtual ‘chat groups’ among Indonesian medical tourists has become a new information platform identified in this study. Some medical tourists stated a specific group chat among experienced medical tourists who had visited Malaysia for medical treatment. The chat group provided suggestions or opinions on the best medical treatment and hospitals in Malaysia, becoming a reliable source of information as most group members visited Malaysia for medical tourism. On the other hand, the repeat visitor’s factors are related to the hospital expertise, the transparency of hospital practice, the quality of prescription drugs, organised administration processes, good hospitality and hospital cleanliness influenced to return to IJN by creating satisfaction with hospital services and treatments.

Decision making often involves a series of complex processes. This study demonstrates the complexity in explaining the decision-making process among medical tourists through the interrelationship between push and pull factors. Diverse factors influenced medical tourists’ decisions in choosing a specific kind of healthcare and its medical destination. For Indonesian medical tourists, the main push factor was to seek quality medical care unavailable in their country. Unsatisfactory experiences with Indonesian hospital practices, including inadequate expertise, inaccurate medical results and unnecessary prescriptions, resulted in increased medical costs, which led to the decision to seek a better medical option in Malaysia. This situation was much influenced by their companions’ preference for them to receive medical treatment in Malaysia. Hence, future research should examine the effect of medical tourist satisfaction and revisit intention for medical services in Malaysia. Satisfied patients tend to spread positive word-of-mouth reviews and recommend them to others. Besides, attention should also be paid to understanding medical tourism from the perspective of service providers. Identifying the advantages, obstacles, opportunities, and threats of providing medical tourism would ensure that first-class services are provided and ongoing business sustainability.

## References

[1] Habibi A, Ariffin AAM (2019). Value as a medical tourism driver interacted by experience quality. Anatolia.

[2] Ratnasari RT, Gunawan S, Pitchay AA, Mohd Salleh MC (2021). Sustainable medical tourism: investigating healthcare travel in Indonesia and Malaysia. Int J Healthcar Manag.

[3] Seow AN, Choong YO, Moorthy K, Chan LM (2017). Intention to visit Malaysia for medical tourism using the antecedents of theory of planned behaviour: a predictive model. Int J Tour Res.

[4] Rahman MK (2019). Medical tourism: tourists’ perceived services and satisfaction lessons from Malaysian hospitals. Tour Rev.

[5] Oktadiana H, Pearce PL (2020). Tourism in Asean cities: features and directions. Routledge handbook of tourism cities.

[6] Pocock NS, Phua KH (2011). Medical tourism and policy implications for health systems: a conceptual framework from a comparative study of Thailand, Singapore and Malaysia. Global Health.

[7] Afthanorhan A, Awang Z, Fazella S (2017). Developing the patients’ loyalty model for medical tourism industry: the case of Malaysia. Int J Soc Syst Sci.

[8] Cham TH, Lim YM, Sia BC, Cheah JH, Ting H (2021). Medical tourism destination image and its relationship with the intention to revisit: a study of Chinese medical tourists in Malaysia. J China Tour Res.

[9] Junio MMV, Kim JH, Lee TJ (2017). Competitiveness attributes of a medical tourism destination: the case of South Korea with importance-performance analysis. J Travel Tour Marke.

[10] Nilashi M, Samad S, Manaf AA, Ahmadi H, Rashid TA, Munshi A (2019). Factors influencing medical tourism adoption in Malaysia: a dematel-fuzzy topsis approach. Comp Indust Engin.

[11] Ormond M (2013). Neoliberal governance and international medical travel in Malaysia.

[12] Rodrigues H, Brochado A, Troilo M, Mohsin A (2017). Mirror, mirror on the wall, who’s the fairest of them all? a critical content analysis on medical tourism. Tour Manag Perspect.

[13] AbuKhalifeh NA, Faller ME (2015). Medical tourism’s impact for health systems: a study from three Asian countries. J Tour Herit Serv Mark.

[14] Kamassi A, Abd Manaf N, Omar A (2020). The identity and role of stakeholders in the medical tourism industry: state of the art. Tourism Rev.

[15] Rahman MK, Zailani S, Musa G (2018). Tourists’ satisfaction and loyalty intention at Shariah compliant private hospitals in Malaysia. Int J Tour Sci.

[16] Yusof H, Rosnan H (2020). The effectiveness of word-of-mouth as a marketing tool in the medical tourism industry in Malaysia: challenges and the way forward. Malays J Qualitative Res.

[17] Chee HL, Barraclough S (2007). Health care in Malaysia: the dynamics of provision, financing and access.

[18] Klijs J, Ormond M, Mainil T, Peerlings J, Heijman W (2016). A state-level analysis of the economic impacts of medical tourism in Malaysia. Asia Pac Econ Lite.

[19] Megeswari M (2018). IJN to have three branches out of KL. The Star.

[20] Medical Travel Today (2018). Spotlight: Sherene Azli, chief executive officer. Malaysia Healthcare Travel Council [Internet].

[21] Malaysia Healthcare Travel Council (2016). Malaysia medical tourism figures 2015 [Internet].

[22] Saragih HS, Jonathan P (2019). Views of Indonesian consumer towards medical tourism experience in Malaysia. J Asia Business Stud.

[23] Malaysia Healthcare Travel Council (2019). Inaugural Malaysia healthcare EXPO 2019 in Jakarta aims to attract 15000 visitors [Internet].

[24] Bernama.com (2019). MHTC boosts promotion for health tourists from Indonesia. Bernama.

[25] Simamora RA, Hendarjatno H (2019). The effects of audit client tenure, audit lag, opinion shopping, liquidity ratio, and leverage to the going concern audit opinion. Asian J Account Res.

[26] Ha J, Yu C, Hwang Y (2021). Analysing the impact of relative push and pull factors on inbound medical tourism in South Korea: focused on BCG matrix applied segment group characteristics. Asia Pacific J Tour Res.

[27] John SP, Larke R (2016). An analysis of push and pull motivators investigated in medical tourism research published from 2000 to 2016. Tour Rev Int.

[28] Ebrahim AH, Ganguli S (2019). A comparative analysis of medical tourism competitiveness of India, Thailand and Singapore. Tourism.

[29] Whittaker A, Lunt N, Horsfall D, Hanefeld J (2015). The implications of medical travel upon equity in lower - and middle - income countries. Handbook on medical tourism and patient mobility.

[30] Ormond M (2015). En route: transport and embodiment in international medical travel journeys between Indonesia and Malaysia. Mobilities.

[31] Ormond M, Sulianti D (2017). More than medical tourism: lessons from Indonesia and Malaysia on south–south intra-regional medical travel. Curr Issues Tour.

[32] Manaf ANH, Maulan S, Hussin H, Kassim PNJ, Alavi R (2017). Service quality, value, satisfaction and future intention in medical tourism. J Tour Hosp Culin Arts.

[33] Ormond M (2015). Solidarity by demand? exit and voice in international medical travel: the case of Indonesia. Soc Sci Med.

[34] Na S, Onn C, Meng C (2016). Travel intentions among foreign tourists for medical treatment in Malaysia: an empirical study. Procedia Soc Behav Sci.

[35] Lim YM, Sia B Medical tourists’ behavioral intention in relation to motivational factors and perceived image of the service providers.

[36] Rahman MK, Sarker M, Hassan A, Hassan A (2021). Medical tourism: the Islamic perspective. Tourism products and services in Bangladesh: concept analysis and development suggestions.

[37] Zailani S, Ali SM, Iranmanesh M, Moghavvemi S, Musa G (2016). Predicting Muslim medical tourists’ satisfaction with Malaysian Islamic friendly hospitals. Tour Manage.

[38] Shukla R, Singh D, Saxena D (2019). Consumer perception of hospitality services in JCI accredited hospitals at Delhi – NCR: An exploratory research on growth of medical tourism. Human Soc Sci Rev.

[39] Musa G, Doshi DR, Wong KM, Thirumoorthy T (2012). How satisfied are inbound medical tourists in Malaysia? a study on private hospitals in Kuala Lumpur. J Travel Tour Mark.

[40] Connell J (2011). Medical tourism.

[41] Jaisuekul M, Teerasukittima C (2017). The study of Thailand ’s cosmetic surgery market and attitudes of surgeons and foreign patients towards cosmetic surgery in Thailand. J Commun Dev Res.

[42] Lydia LG, Frederick JR (2015). Medical tourism: consumers’ concerns over risk and social challenges. J Travel Tour Mark.

[43] Gopalan N, Noor MS, Mohamed SM (2021). The pro-medical tourism stance of Malaysia and how it affects stem cell tourism industry. SAGE Open.

[44] Cohen E, Cohen E (2008). Medical tourism in Thailand. Explorations in Thai tourism.

[45] Connell J, Lunt N, Horsfall D, Hanefeld J (2015). Medical tourism: concepts and definitions. Handbook on medical tourism and patient mobility.

[46] Hanefeld J, Lunt N, Smith R, Horsfall D (2015). Why do medical tourists travel to where they do? The role of networks in determining medical travel. Soc Sci Med.

[47] Kim S, Arcodia C, Kim I (2019). Critical success factors of medical tourism: the case of South Korea. Int J Environ Res Public Health.

[48] Laugesen MJ, Vargas-Bustamante A (2010). A patient mobility framework that travels: European and United States–Mexican comparisons. Health Policy.

[49] Daily News (2018). 700,000 medical tourists visited Turkey in 2017. Daily News.

[50] International Atomic Energy Agency (2017). Fiji seeks to establish a radiotherapy facility to improve cancer treatment [Internet].

[51] Pollard K (2015). When governments fund medical tourism: the rewards are significant [Internet]. International Medical Travel Journal (IMTJ).

[52] Yadav H, Christian A, Teguh PK, Robin G (2017). The health care system in Malaysia. Health care systems in developing countries in Asia.

[53] Habibu S (2021). Malaysia is top international destination for medical tourism. The Star.

[54] Phua KL (2016). The promotion of cross-border medical tourism in developing countries: economic growth at the expense of healthcare system efficiency and cost containment?. Open Public Health J.

[55] Malaysia Healthcare Travel Council (MHTC) (2019). Malaysia’s medical tourism on the high [Internet].

[56] Cham T, Lim Y, Sia B, Cheah J, Ting H (2020). Medical tourism destination image and its relationship with the intention to revisit: a study of Chinese medical tourists in Malaysia. J China Tour Res.

[57] Rahman MK, Zailani S, Musa G (2017). Tapping into the emerging Muslim-friendly medical tourism market: evidence from Malaysia. J Islam Mark.

[58] Chee HL, Whittaker A (2019). Moralities in international medical travel: moral logics in the narratives of Indonesian patients and locally-based facilitators in Malaysia. J Ethn Migr Stud.

[59] Jaapar M, Musa G, Moghavvemi S, Saub R (2017). Dental tourism: Examining tourist profiles, motivation and satisfaction. Tourism Manage.

[60] Ormond M, Mun WK, Khoon CC (2014). Medical tourism in Malaysia: How can we better identify and manage its advantages and disadvantages?. Glob Health Action.

[61] Zain NAM, Zahari MSM, Hanafiah MH, Zulkifly MI (2017). Medical tourism: tourist information sources, satisfaction and post behavioral. J Tourism Hosp Culin Arts.

[62] Garg D, Batra R, Banerji A (2020). Low cost, quality treatment and excellent hospitality makes India the best destination for medical tourism. Int J Innovative Res Med Sci.

[63] Ancy RJ, Shenoy RP, Jodalli PS, Pasha IM (2020). Benefits of medical and dental tourism: a review. J Dental Med Sci.

[64] European Commission (2014). Journalist workshop on organ donation and transplantation: recent facts & figures.

[65] Lamoree L (2013). Medical tourism development in Cebu City; effects on local development and the local population. Master’s thesis.

[66] Turner L (2007). First world health care at third world prices: globalisation, bioethics and medical tourism. BioSocieties.

[67] Lwin H, Punnakitikashem P, Thananusak T (2021). The level and determinants of international patient satisfaction with dental tourism in Bangkok, Thailand. Cogent Busi Manage.

[68] Agbabiaka HI, Omisore EO, Odunsi O (2017). Medical tourism in Nigeria: a multivariate analysis of challenges faced by patrons. Int J Tourism Cities.

[69] Cohen IG (2010). Protecting patients with passports: medical tourism and the patient-protective argument. Iowa Law Rev.

[70] Zolfagharian M, Rajamma RK, Naderi I (2018). Determinants of medical tourism destination selection process. J Hosp Mark Manage.

[71] Pricewaterhouse Coopers (2016). Restoring trust to Nigeria’s healthcare system. Pricewaterhouse Coopers (PwC) report.

[72] Bochaton A (2014). Cross-border mobility and social networks: Laotians seeking medical treatment along the Thai border. Soc Sci Med.

[73] Moghadam F, Masoudi Asl I, Hessam S, Farahani M (2020). In search a medical tourism marketing pattern in Iran: the case of cultural sensitivities. Int J Healthc Manag.

[74] Mathijsen A, Mathijsen F (2020). Diasporic medical tourism: a scoping review of quantitative and qualitative evidence. Global Health.

[75] Connell J (2016). Reducing the scale? From global images to border crossings in medical tourism. Global Networks.

[76] Lee JY, Kearns RA, Friesen W, Lunt N, Horsfall D, Hanefeld J (2015). Diasporic medical return: Korean immigrants’ use of homeland medical services. Handbook on medical tourism and patient mobility.

[77] Lunt N, Smith R, Exworthy M, Green S, Horsfall D, Mannion R (2011). Medical tourism: treatments, markets and health system implications: a scoping review.

[78] Whittaker A (2015). ‘Outsourced’ patients and their companions: Stories from forced medical travellers. Global Public Health.

[79] Verra SE, Kroeze R, Ruggeri K (2016). Facilitating safe and successful cross-border healthcare in the European Union. Health Policy.

[80] Cohen IG (2012). Circumvention tourism. Cornell Law Rev.

[81] Cohen IG, Lunt N, Horsfall D, Hanefeld J (2015). Medical tourism for services illegal in patients ’ home country. Handbook on medical tourism and patient mobility.

[82] Piersanti V, Consalvo F, Signore F, Del Rio A, Zaami S (2021). Surrogacy and ‘procreative tourism’. What does the future hold from the ethical and legal perspectives?. Medicina.

[83] Lozanski K (2015). Transnational surrogacy: Canada’s contradictions. Soc Sci Med.

[84] Hoof V, Pennings G, Sutter DP (2015). Cross-border reproductive care for law evasion: a qualitative study into the experiences and moral perspectives of French women who go to Belgium for treatment with donor sperm. Soc Sci Med.

[85] Ormond ME (2020). International medical travel, or medical tourism. Int Encyclopedia of Human Geography.

[86] Zolfagharian M, Rajamma RK, Naderi I (2018). Determinants of medical tourism destination selection process. Journal of Hospitality Marketing & Management.

[87] Fetscherin M, Stephano RM (2016). The medical tourism index: Scale development and validation. Tourism Management.

[88] Khan MJ, Chelliah S, Haron MS, Ahmed S (2017). Push factors, risks, and types of visit intentions of international medical travelers–A conceptual model. International Journal of Healthcare Management.

[89] Smith PC, Forgione DA (2007). Global outsourcing of healthcare: A medical tourism decision model. Journal of Information Technology Case and Application Research.

[90] Heung VCS, Kucukusta D, Song H (2011). Medical tourism development in Hong Kong: An assessment of the barriers. Tourism Management.

[91] Connell J (2015). From medical tourism to transnational health care? An epilogue for the future. Social Science and Medicine.

